# Five steps to solving the vaccine inequity crisis

**DOI:** 10.1371/journal.pgph.0000032

**Published:** 2021-10-13

**Authors:** Tedros Adhanom Ghebreyesus

**Affiliations:** World Health Organization, Geneva, Switzerland; PLOS Global Public Health, UNITED STATES

We can end the acute of stage of this pandemic very quickly. Highly safe and effective vaccines have, for the moment at least, significantly weakened the link between cases and death in the countries with the highest vaccine coverage [1]. This has allowed those countries, which have so far monopolized vaccine supply, to open up and enjoy a certain level of economic recovery [2].

The picture in most low- and lower middle-income however is far bleaker. The 30 poorest countries in the world have fully vaccinated approximately just 2% of their populations [1]. This is not even enough to vaccinate health workers who have been on the frontline of this pandemic, nor those at greatest risk. Compounded by the more transmissible Delta variant, this is translating to avoidable deaths, causing health systems to collapse and undermining the delivery of essential health services.

A year ago, the World Health Organization (WHO) warned that vaccine nationalism would only prolong the pandemic and offered a number of ways for governments and companies to ensure the equitable manufacture and sharing of vaccine doses [3], including COVAX, a unique mechanism designed to pool the risks of developing vaccines, and their procurement. The two-track pandemic we are now witnessing could have been avoided but instead is being exacerbated. Some countries that have vaccinated the majority of their populations have already begun rolling out booster shots and some are also developing vaccine stockpiles [4]. Even vaccines produced in Africa have been shipped to countries that have already vaccinated the majority of their populations [5].

The multilateral leads taskforce on COVID-19, which includes the International Monetary Fund, the World Bank Group, the World Trade Organization and WHO, meets regularly to identify ways to solve the vaccine crisis and accelerate vaccine rollout to those that have so far only received sparse supply.

To end the acute stage of this pandemic by protecting the most vulnerable and minimizing deaths, I want all countries to commit to the eminently feasible target of at least 40% vaccination coverage of their total population by the end of this year. It’s more than possible, not least because of recent projections [4] that highly vaccinated countries currently have 300 million stockpiled vaccines and this will be more than a billion by the end of the year. At a time of vaccine scarcity in the African continent and many other low and lower middle-income countries, this is morally repugnant and epidemiological madness.

At the time of writing, close to 10,000 people were recorded as dying every single day because of this virus [6]. We can end this crisis, but only with the support of key countries and vaccine manufacturers. There are five simple steps that if implemented now would save a lot of lives.

First, those countries that have contracted high volumes of vaccines should swap near-term delivery schedules with COVAX and the African Vaccine Acquisition Trust (AVAT). These mechanisms work but have been underused. At a time of global vaccine scarcity, stockpiled vaccines will ironically make populations less safe by allowing the virus to rip through unvaccinated communities, giving it free rein to potentially mutate into a variant that can evade vaccines.

Second, vaccine manufacturers should immediately prioritize and fulfill their contracts to COVAX and AVAT, and provide regular, clear supply forecasts. The lack of transparency or accountability around vaccine contracts has led to poorer countries sometimes paying more for vaccines than richer countries [7]. It’s clear that in future the world will need an agreed global mechanism for speeding up the development of global public goods such as vaccines, and their equitable manufacturing and distribution, in the event of an emergency. We expect such a mechanism to be part of discussions about the new global health security architecture, including when WHO Member States convene for a Special Session of the World Health Assembly in November, to discuss the idea of a treaty or other international agreement for pandemic preparedness and response.

Third, G7 and all dose-sharing countries must fulfill their pledges urgently, with enhanced pipeline visibility, sufficient product shelf life and support for supplementary supplies. High-income countries have promised to donate more than 1 billion doses, but less than 15% of those have materialized so far [8].

Fourth, all countries must eliminate export restrictions and any other trade barriers on COVID-19 vaccines and the inputs involved in their production. The pandemic has exploited cracks in global solidarity and structural inequalities, with hoarding of personal protective equipment, tests, treatments, and vaccines. If we don’t change course, that pattern won’t change. It’s not only a moral failure, it’s epidemiologically and economically self-defeating and is having knock-on effects on everything from food prices [9] to gender equality and even fostering national and regional insecurity.

Finally, getting this right takes leadership, economic capital, and a realization that for a fast-moving respiratory pathogen, the only way out is to do so together. It is promising that some countries have recently increased their sharing of vaccine doses [10] globally but there needs to be more and it needs to happen faster. Furthermore, on the manufacturing side, since the beginning of the outbreak WHO has called for the sharing of licenses, technology and know-how.

Most manufacturers have largely spurned the opportunities to share technology and know-how and public health-oriented licensing, despite a number of mechanisms being set up including the COVID-19 Technology Access Pool and the mRNA vaccine technology transfer hub [11], which is now moving ahead in South Africa. The sluggish response at the heart of a pandemic is ill-judged and reminiscent of similar mistakes made during the peak of the HIV crisis. That bitter experience is in large part why the push for a temporary waiver in intellectual property has now been backed by the majority of countries and remains on the table at the World Trade Organization.

As world leaders meet at the G20 Summit in Rome, there is an opportunity to outline a comprehensive plan to end the acute stage of this pandemic everywhere. The Delta variant has reinforced just how interconnected the world is and that new variants emerging anywhere are a threat everywhere. The best way to protect people is to prioritise protecting everyone. The time for commitments has passed; the time to deliver is now.
